# Effects of evidence-based nursing under a quantitative assessment strategy on cancer-related fatigue, self-management ability, and quality of life in lung cancer patients undergoing chemotherapy

**DOI:** 10.3389/fonc.2025.1685591

**Published:** 2025-12-04

**Authors:** Liwei Zhang, Haixian Fang, Yongqian Zhang, Xin Su, Juan Zhang, Li He, Changwen Bo, Yusha Yan, Jing Liu, Feifei Wang

**Affiliations:** Department of Oncology, The First Hospital of Hebei Medical University, Shijiazhuang, Hebei, China

**Keywords:** evidence-based nursing, quantitative assessment strategy, lung cancer, chemotherapy, cancer-related fatigue

## Abstract

**Objective:**

To investigate the effects of evidence-based nursing under a quantitative assessment strategy (EB-NQAS) on cancer-related fatigue (CRF), self-management ability, quality of life (QoL), and adverse events in lung cancer patients undergoing chemotherapy.

**Methods:**

A single-center, prospective, randomized controlled trial (RCT) was conducted, enrolling 150 lung cancer patients undergoing chemotherapy between January 2024 and January 2025. Participants were randomized into the intervention group (n=75) and control group (n=75) using a random number table. The intervention group received EB-NQAS, which featured personalized care plans based on symptom quantification via the Edmonton Symptom Assessment Scale (ESAS), while the control group received routine nursing care. Outcomes were assessed at baseline and 3 months. CRF was assessed using the Piper Fatigue Scale (PFS), self-management ability via the Adult Health Self-Management Scale (AHSMSRS), and QoL via the European Organization for Research and Treatment of Cancer Quality of Life Core Questionnaire (EORTC-QLQ-C30). Adverse events were recorded. Data were analyzed using linear mixed-effects models and chi-square tests.

**Results:**

Analyses were conducted on all 150 randomized participants per the intention-to-treat principle. After 3 months, the EB-NQAS group showed a significantly greater reduction in the total PFS score compared to the control group (22.88 ± 1.72 vs. 25.36 ± 1.63; group × time interaction, P<0.001). The intervention group also demonstrated significantly greater improvements in total AHSMSRS scores (146.00 ± 9.77 vs. 128.45 ± 12.45) and global health status/QoL scores (77.66 ± 9.74 vs. 68.91 ± 9.51) than the control group (all group × time interactions, P<0.001). The incidence of gastrointestinal reactions in the intervention group (5.33%) was lower than that in the control group (18.67%, P = 0.012). No significant differences were observed in other adverse events (P>0.05).

**Conclusion:**

Evidence-based nursing under a quantitative assessment strategy effectively reduces CRF, improves self-management ability and QoL, and decreases gastrointestinal reactions in lung cancer patients undergoing chemotherapy, demonstrating strong potential for clinical application.

## 1 Introduction

Lung cancer remains a leading global health threat, characterized by high incidence and mortality rates. According to recent epidemiological data (1, 2), there are approximately 2.2 million new lung cancer cases and 1.8 million cancer-related deaths worldwide annually. In China, the burden is particularly severe, with an estimated 820,000 new cases and 710,000 deaths each year (3). Over recent decades, the incidence of lung cancer has shown a steady upward trend, with a notable shift toward younger onset. Owing to its insidious clinical presentation, most patients are diagnosed at advanced stages, which, combined with its high morbidity and mortality, imposes significant psychological distress on patients and their families.

Chemotherapy is a cornerstone in the management of lung cancer, yet it is frequently complicated by treatment-related adverse effects, particularly cancer-related fatigue (CRF). CRF is a multidimensional and distressing symptom that not only impairs patients’ quality of life (QoL) but may also compromise treatment adherence and prognosis (4). This symptom burden not only diminishes QoL but also impairs a patient’s capacity for self-management, creating a vicious cycle of functional decline and psychological distress (5). Conventional nursing care, however, is often reactive, focusing on addressing existing symptoms rather than proactively preventing complications. This approach is limited by its narrow focus on isolated issues, failing to holistically address the multifactorial needs of patients, including psychological, social, and physiological dimensions.

Effective nursing interventions must therefore address these interconnected issues holistically. Evidence-based nursing (EBN) provides a framework for integrating the best research evidence with clinical expertise and patient values to improve outcomes (6), such as enhanced treatment compliance and quality of life (QoL) in lung cancer patients (7). Despite the critical role of nursing in supportive oncology, conventional reactive strategies have proven insufficient in effectively mitigating cancer-related fatigue (CRF) and improving QoL among chemotherapy-treated lung cancer patients. This insufficiency persists even as EBN expands rapidly to bridge evidence-to-practice gaps in symptom management (8), emphasizing individualized care plans derived from clinical research, practitioner experience, and patient preferences (9). There remains an unmet need for innovative interventions that can systematically assess symptoms, integrate evidence-based practices (7), and dynamically adjust care to meet patients’ evolving needs—addressed through initiatives like structured educational programs (e.g., SEEK™) that operationalize EBN principles in real-world oncology settings (10).

To address this gap, our team developed a novel intervention protocol—evidence-based nursing under a quantitative assessment strategy (EB-NQAS). This approach utilizes the Edmonton Symptom Assessment Scale (ESAS) to quantitatively evaluate nine key symptom dimensions (e.g., pain, fatigue, nausea) in lung cancer patients undergoing chemotherapy. By integrating ESAS results with clinical data, it enables the formulation of personalized care plans (e.g., graded pain management, fatigue intervention, nausea prevention, and psychological support) based on best evidence from evidence-based medicine, which has been shown to improve patient outcomes through enhanced symptom monitoring and tailored interventions (11, 12). These plans are dynamically adjusted during chemotherapy to optimize symptom control and QoL, a strategy supported by research showing the value of routine symptom monitoring (13). The novelty of this approach lies not in the use of EBN or symptom scales alone, but in their structured integration into a dynamic, cyclical process: quantitative assessment actively guides the selection of evidence-based interventions, and subsequent assessments evaluate their efficacy, allowing for real-time adjustments. This study, therefore, hypothesizes that patients receiving the EB-NQAS intervention (1) will report significantly lower levels of cancer-related fatigue (2), will demonstrate significantly improved self-management ability, and (3) will have a significantly higher quality of life compared to patients receiving routine nursing care.

## 2 Methods

### 2.1 Study design and participants

This was a single-center, prospective, parallel-group, randomized controlled trial (RCT) conducted at The First Hospital of Hebei Medical University from January 2024 to January 2025. The trial followed the Consolidated Standards of Reporting Trials (CONSORT) 2025 guidelines (14) and was registered with ClinicalTrials.gov (Identifier: NCT07049237).

Patients meeting the eligibility criteria were enrolled. Inclusion Criteria: ① Pathologically or radiologically confirmed lung cancer (World Health Organization [WHO] 2021 diagnostic criteria); ② Scheduled to receive first-line chemotherapy (platinum-based regimen); ③ Eastern Cooperative Oncology Group (ECOG) performance status of 0-2; ④ Estimated survival time >6 months (assessed by treating oncologist); ⑤ Normal mental and cognitive function (Mini-Mental State Examination [MMSE] score (15) ≥24); ⑥ Voluntary participation and signed informed consent. This study focused on patients receiving first-line chemotherapy to ensure a homogeneous population at a similar point in their treatment trajectory, thereby minimizing the confounding effects of prior therapies on fatigue, QoL, and self-management ability. Exclusion Criteria: ① Concurrent severe organ dysfunction (Child-Pugh score (16) ≥B for liver; estimated glomerular filtration rate [eGFR] <30 mL/min/1.73m² for kidney); ② Active neurological disorders (e.g., stroke, epilepsy) affecting symptom reporting; ③ Acute infectious diseases (e.g., tuberculosis with sputum positivity); ④ Known allergies to chemotherapy drugs (e.g., platinum agents); ⑤ Acute illnesses requiring hospitalization (e.g., pneumonia, myocardial infarction); ⑥ Secondary malignancies (pathologically confirmed); ⑦ Chemotherapy contraindications (e.g., uncontrolled heart failure); ⑧ Inability to communicate in the local language (assessed via nurse interview); ⑨ Concurrent radiotherapy or immunotherapy; ⑩ Participation in other interventional clinical trials within 3 months.

#### 2.1.1 Sample size calculation

Sample size was calculated using G*Power 3.1.9.7 software. Based on a repeated measures analysis with two groups and two primary time points (baseline and 3 months), we aimed to detect a medium effect size (f = 0.25) for the group × time interaction on the primary outcome (CRF total score). With a two-tailed α=0.05, power=0.80, and a correlation of 0.5 between repeated measures, a total sample size of 128 participants (64 per group) was required. To account for an anticipated dropout rate of approximately 15%, we aimed to recruit 150 participants (75 per group).

#### 2.1.2 Randomization and allocation concealment

Eligible participants were randomly assigned (1:1) to the intervention or control group using a computer-generated random number sequence. Allocation sequences were generated by a statistician not involved in participant recruitment or data collection. Sequentially numbered, sealed, opaque envelopes were used to conceal allocation until the point of enrollment, ensuring that the research team was unaware of group assignment during the screening and consent process.

### 2.2 Interventions

A dedicated nursing team (1 oncologist, 1 head nurse with 10+ years of oncology experience, 2 senior nurses [5+ years], and 4 certified oncology nurses) was formed and underwent a 4-week training program before participant enrollment. Training covered the study protocol, standardized administration of assessment tools (ESAS, PFS), and delivery of the intervention components.

#### 2.2.1 Control group: routine nursing care

Participants in the control group received standard nursing care as per the hospital’s oncology nursing guidelines (version 2023). This care was primarily reactive and included:

- Health education: Upon admission, nurses provided general verbal and written information about lung cancer, chemotherapy procedures, common side effects, and the importance of treatment adherence.
- Symptom management: When patients reported symptoms (e.g., nausea, pain), nurses provided standard supportive care, such as administering prescribed PRN (as-needed) antiemetics or analgesics and offering general advice (e.g., “eat small, frequent meals,” “get adequate rest”).
- Psychological support: Nurses provided brief, informal emotional support during routine interactions. No structured psychological assessment or intervention was provided.
- Dietary guidance: A standard hospital diet was provided. General dietary advice was given upon request.

#### 2.2.2 Group: evidence-based nursing under quantitative assessment strategy

In addition to routine care, participants in the intervention group received the structured EB-NQAS protocol, which was delivered through one-on-one, in-person sessions by the trained nursing team. The intervention cycle was aligned with the chemotherapy schedule and involved the following core procedures:

1. Baseline Assessment (within 24 hours of admission): A trained nurse administered the Edmonton Symptom Assessment Scale (ESAS) to quantify the severity of 9 common symptoms on a 0–10 scale. This was coupled with a review of clinical data (tumor stage, chemotherapy regimen).
2. Tailored Care and Self-Management Planning (initiated within 48 hours): Based on the ESAS scores, the nurse collaborated with the patient to develop a personalized care and self-management plan. Evidence-based pathways were triggered by specific score thresholds:
  - Pain Management: For ESAS pain score ≥4, a graded algorithm was initiated. Mild Pain (4–6): Nurses taught and supervised non-pharmacological methods like guided imagery and progressive muscle relaxation (20 min, twice daily). Moderate-to-Severe Pain (≥7): Nurses alerted the oncologist to initiate or adjust analgesic prescriptions according to WHO guidelines and provided education on managing breakthrough pain. • Fatigue Intervention: For ESAS fatigue score ≥5, a structured energy conservation and activity plan was developed. This included education on balancing rest and activity, and setting a daily goal for light exercise (10–15 minutes of walking), monitored with a patient diary.
  - Nausea/Vomiting Prevention: For ESAS nausea score ≥3, in addition to standard prophylactic antiemetics, nurses provided targeted dietary counseling (avoiding greasy/spicy foods, eating small, cool meals) and taught acupressure techniques (on the P6 point) for self-administration before and during chemotherapy.
  - Psychological Support: For ESAS depression or anxiety score ≥5, nurses initiated weekly, 20-minute structured supportive conversations focused on active listening, validating feelings, and teaching simple cognitive reframing techniques (identifying and challenging catastrophic thoughts).
  - Self-Management Enhancement: Across all domains, patients were educated on how to monitor their symptoms using a simplified daily diary, set weekly goals for self-care behaviors, and identify when to report worsening symptoms to the nursing staff.
3. Dynamic Evaluation and Adjustment: ESAS was re-administered daily during chemotherapy infusions and weekly between cycles. If a symptom score failed to improve or worsened, the nursing team met to review the care plan, consult updated evidence (via UpToDate), and adjust the interventions accordingly in collaboration with the patient and medical team.

### 2.3 Measures

#### 2.3.1 Primary outcome

##### 2.3.1.1 Cancer-related fatigue

CRF was assessed using the Piper Fatigue Scale (PFS) (17). The PFS is a 22-item instrument that evaluates four dimensions of subjective fatigue: behavioral/severity, affective/meaning, sensory, and cognitive/mood. Items are scored on a 0–10 scale, with higher total scores (range 0–220) indicating more severe fatigue. The scale has demonstrated excellent internal consistency (Cronbach’s α=0.92) and validity in oncology populations (18).

#### 2.3.2 Secondary outcomes

##### 2.3.2.1 Self-management ability

This was evaluated using the Adult Health Self-Management Scale (AHSMSRS) (19). This 38-item scale measures three domains: self-management environment, behavior, and cognition. Items are rated on a 5-point Likert scale (1–5), with total scores ranging from 38 to 190. Higher scores reflect better self-management ability. It has been validated in chronic illness populations, showing good reliability (Cronbach’s α=0.89).

##### 2.3.2.2 Quality of life

QoL was measured using the European Organization for Research and Treatment of Cancer Quality of Life Core Questionnaire (EORTC-QLQ-C30, version 3.0) (20). This 30-item questionnaire includes five multi-item functional scales (physical, role, emotional, cognitive, social), three multi-item symptom scales (fatigue, nausea/vomiting, pain), several single-item symptom measures, and a global health status/QoL scale. Scores are transformed to a 0–100 scale. Higher scores on the functional and global QoL scales represent better functioning/QoL, whereas higher scores on the symptom scales represent a higher level of symptomatology. The EORTC-QLQ-C30 is well-validated in lung cancer patients (Cronbach’s α≥0.85 for most scales) (20, 21).

##### 2.3.2.3 Adverse events

The incidence and severity of chemotherapy-related adverse effects (gastrointestinal reactions, hepatic dysfunction, myelosuppression, neurotoxicity, alopecia) were monitored and graded throughout the study by the clinical team using the National Cancer Institute’s Common Terminology Criteria for Adverse Events (CTCAE) v5.0.

### 2.4 Study procedures

Following informed consent, baseline data, including demographic information and all outcome measures (PFS, AHSMSRS, EORTC-QLQ-C30), were collected by trained research assistants who were blinded to group allocation. Participants were then randomized. The intervention or control nursing protocols were implemented from the first day of chemotherapy and continued for a 3-month period. All outcome measures were re-administered at the 3-month follow-up visit by the same blinded research assistants.

### 2.5 Fidelity of intervention

Intervention fidelity was monitored throughout the study. A checklist derived from the EB-NQAS protocol was used by a senior research nurse to conduct monthly audits of 10% of intervention sessions. Nurse logbooks and patient diaries were also reviewed. Provider adherence to the protocol was high, with a mean of 96% of all scheduled intervention components delivered as planned across all encounters. Patient adherence was also strong, with participants completing an average of 94% of their assigned self-care activities (e.g., daily walking, dietary logs).

### 2.6 Statistical analysis

Data were analyzed using SPSS Statistics version 27.0 (IBM Corp., Armonk, NY, USA). All analyses followed the intention-to-treat (ITT) principle, including all 150 randomized participants. Missing data for longitudinal outcomes were handled using the full information maximum likelihood estimation method inherent in mixed-effects models. A two-sided P-value < 0.05 was considered statistically significant. Baseline demographic and clinical characteristics were compared between groups using independent t-tests for continuous variables and chi-square tests (or Fisher’s exact test where appropriate) for categorical variables. For longitudinal outcomes (PFS, AHSMSRS, and EORTC-QLQ-C30 scores), linear mixed-effects models were used to assess the intervention effect over time. These models included fixed effects for group (intervention vs. control), time (baseline vs. 3 months), and the group × time interaction, with a random intercept for each participant to account for intra-individual correlation. The primary analysis focused on the significance of the group × time interaction term. To further assess the clinical impact at the study endpoint, independent t-tests were used to compare the absolute scores between groups at the 3-month follow-up. Incidence rates of adverse events were compared between groups using chi-square or Fisher’s exact tests.

### 2.7 Ethical considerations

The study protocol was approved by the Institutional Review Board (IRB) of The First Hospital of Hebei Medical University and was conducted in compliance with the Declaration of Helsinki. All participants provided written informed consent prior to any study-related procedures.

## 3 Results

### 3.1 Participant flow and baseline characteristics

**Figure 1:**
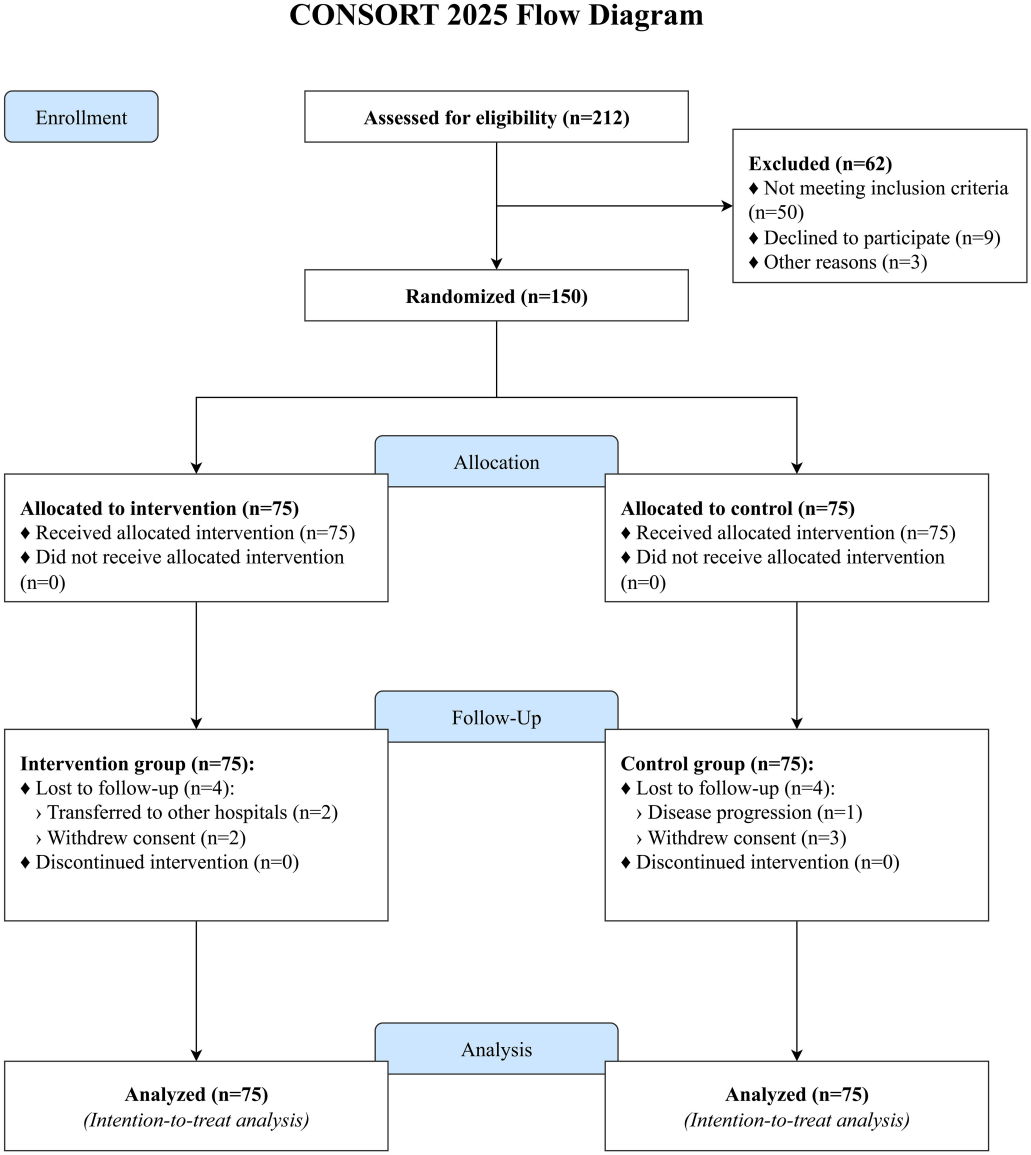
CONSORT 2025 flow diagram of participant enrollment, allocation, and follow-up.

A total of 212 patients were screened for eligibility, of whom 150 were randomized (75 to the intervention group and 75 to the control group). During the 3-month follow-up period, 4 participants in the intervention group were lost to follow-up (2 transferred to other hospitals, 2 withdrew consent), and 4 in the control group were lost to follow-up (1 due to disease progression, 3 withdrew consent). The final analysis included all 150 randomized participants (75 per group), consistent with the ITT principle (**Figure 1**). Baseline demographic and clinical characteristics were well-balanced between the two groups, with no statistically significant differences observed (all P > 0.05), ensuring comparability (**Table 1**). The most common chemotherapy regimens were platinum-based doublets, including cisplatin/carboplatin with pemetrexed for adenocarcinoma and with etoposide for small cell carcinoma. Patients with early-stage disease (Stage I-III) received chemotherapy in an adjuvant or neoadjuvant setting.

**Table 1:** Baseline demographic and clinical characteristics of the two groups.

Indicator	Control group (n=75)	Intervention group (n=75)	χ²/t	P
Age (years), mean ± SD	53.21 ± 11.09	53.83 ± 10.91	0.345	0.731
BMI (kg/m²), mean ± SD	22.94 ± 3.25	22.69 ± 3.41	-0.460	0.647
Gender (male/female), n (%)	48 (64.0) / 27 (36.0)	45 (60.0) / 30 (40.0)	0.255	0.614
Disease duration (months), mean ± SD ^a^	4.76 ± 1.23	4.32 ± 1.57	-1.911	0.058
Smoking, n (%)	32 (42.7)	36 (48.0)	0.535	0.465
Clinical stage ^b^, n (%)			0.689	0.876
- Stage I	5 (6.7)	7 (9.3)	–	–
- Stage II	8 (10.7)	10 (13.3)	–	–
- Stage III	30 (40.0)	28 (37.3)	–	–
- Stage IV	32 (42.7)	30 (40.0)	–	–
Pathological type, n (%)			0.164	0.921
- Small cell carcinoma	19 (25.3)	19 (25.3)	–	–
- Squamous cell carcinoma	39 (52.0)	37 (49.3)	–	–
- Adenocarcinoma	17 (22.7)	19 (25.3)	–	–
Chemotherapy regimen, n (%)			0.451	0.798
- Cisplatin/Carboplatin + Pemetrexed	33 (44.0)	35 (46.7)	–	–
- Cisplatin/Carboplatin + Gemcitabine	23 (30.7)	21 (28.0)	–	–
- Cisplatin/Carboplatin + Etoposide	19 (25.3)	19 (25.3)	–	–
Comorbidities, n (%)				
- COPD	17 (22.7)	15 (20.0)	0.073	0.787
- Hypertension	9 (12.0)	10 (13.3)	0.059	0.807
- Diabetes	18 (24.0)	16 (21.3)	0.152	0.697

a Disease duration refers to the time from initial diagnosis to study enrollment.

b Patients with early-stage disease (Stage I-III) received chemotherapy in an adjuvant or neoadjuvant setting.

### 3.2 Cancer-related fatigue outcomes

At baseline, there were no significant differences in CRF scores between the two groups (**Table 2**). At 3 months post-intervention, the intervention group reported lower mean scores across all CRF domains. The linear mixed-effects model revealed a significant group × time interaction effect for the total PFS score, indicating that the change in fatigue over time was significantly different between the two groups. Significant interaction effects were also found for all subscales (**Table 2**). Furthermore, at the 3-month follow-up, the total PFS score in the intervention group was significantly lower than in the control group (22.88 ± 1.72 vs. 25.36 ± 1.63, P < 0.001).

**Table 2:** Comparison of cancer-related fatigue scores between the two groups.

Indicator	Time	Control group (n=75)	Intervention group (n=75)	Group × time Interaction		P-value at 3 months
		Mean ± SD	Mean ± SD	F(1, 148)	P	
Behavioral	Pre-intervention	4.87 ± 0.41	4.81 ± 0.44	53.92	<0.001	<0.001
	3 months post-intervention	5.76 ± 0.39	5.31 ± 0.36			
Emotional	Pre-intervention	5.02 ± 0.45	5.06 ± 0.48	18.84	<0.001	<0.001
	3 months post-intervention	6.39 ± 0.49	6.06 ± 0.44			
Cognitive	Pre-intervention	5.67 ± 0.49	5.69 ± 0.45	39.29	<0.001	<0.001
	3 months post-intervention	6.34 ± 0.41	5.91 ± 0.43			
Physical fatigue	Pre-intervention	5.32 ± 0.37	5.23 ± 0.39	37.34	<0.001	<0.001
	3 months post-intervention	6.87 ± 0.43	6.41 ± 0.49			
**Total score**	**Pre-intervention**	**20.88 ± 1.72**	**20.79 ± 1.68**	**81.54**	**<0.001**	**<0.001**
	**3 months post-intervention**	**25.36 ± 1.63**	**22.88 ± 1.72**			

c P-value from independent t-test comparing scores between groups at the 3-month follow-up.Bold values indicate P < 0.05.

### 3.3 Self-management ability outcomes

Baseline self-management ability scores were similar between groups (**Table 3**). After 3 months, the intervention group demonstrated greater improvements in self-management scores compared with the control group. A significant group × time interaction effect was found for the total AHSMSRS score, as well as for all subscales (**Table 3**). At the 3-month follow-up, the intervention group had a significantly higher total AHSMSRS score than the control group (146.00 ± 9.77 vs. 128.45 ± 12.45, P < 0.001).

**Table 3:** Comparison of self-management ability scores between the two groups.

Indicator	Time	Control group (n=75)	Intervention group (n=75)	Group × time Interaction		P-value at 3 months
		Mean ± SD	Mean ± SD	F (1, 148)	P	
Self-management environment	Pre-intervention	22.37 ± 4.02	22.43 ± 4.13	90.93	<0.001	<0.001
	3 months post-intervention	33.01 ± 4.11	39.31 ± 3.98			
Self-management behavior	Pre-intervention	34.87 ± 4.09	34.79 ± 4.51	13.21	<0.001	0.002
	3 months post-intervention	47.09 ± 4.16	49.65 ± 4.46			
Self-management cognition	Pre-intervention	35.04 ± 4.36	35.09 ± 4.29	156.38	<0.001	<0.001
	3 months post-intervention	48.35 ± 4.18	57.04 ± 4.33			
**Total score**	**Pre-intervention**	**92.28 ± 12.47**	**92.31 ± 12.93**	**92.24**	**<0.001**	**<0.001**
	**3 months post-intervention**	**128.45 ± 12.45**	**146.00 ± 9.77**			

c P-value from independent t-test comparing scores between groups at the 3-month follow-up.Bold values indicate P < 0.05.

### 3.4 Quality of life outcomes

There were no significant differences in baseline QoL scores between the groups (**Table 4**). At the 3-month follow-up, the intervention group reported higher mean scores on all QoL functional scales. Analysis with the linear mixed-effects model showed a significant group × time interaction for the Global Health Status/QoL scale. Significant interaction effects favoring the intervention group were also observed for all functional scales (**Table 4**). The mean Global Health Status/QoL score at 3 months was significantly higher in the intervention group compared to the control group (77.66 ± 9.74 vs. 68.91 ± 9.51, P < 0.001).

**Table 4:** Comparison of quality of life scores between the two groups.

Indicator	Time	Control group (n=75)	Intervention group (n=75)	Group × Time Interaction		P-value at 3 months ^c^
		Mean ± SD	Mean ± SD	F (1, 148)	P	
Physical functioning	Pre-intervention	57.32 ± 9.38	57.46 ± 9.76	26.26	<0.001	<0.001
	3 months post-intervention	68.43 ± 9.01	76.32 ± 9.83			
Social functioning	Pre-intervention	65.03 ± 8.74	65.73 ± 8.87	24.39	<0.001	<0.001
	3 months post-intervention	71.34 ± 8.65	78.43 ± 8.93			
Emotional functioning	Pre-intervention	63.28 ± 9.34	63.23 ± 10.04	22.26	<0.001	<0.001
	3 months post-intervention	70.03 ± 9.04	77.43 ± 10.14			
Role functioning	Pre-intervention	58.61 ± 10.04	58.94 ± 10.76	52.1	<0.001	<0.001
	3 months post-intervention	65.82 ± 11.34	78.46 ± 10.07			
**Global Health Status / QoL**	**Pre-intervention**	**59.81 ± 9.38**	**60.09 ± 9.86**	**30.01**	**<0.001**	**<0.001**
	**3 months post-intervention**	**68.91 ± 9.51**	**77.66 ± 9.74**			

c P-value from independent t-test comparing scores between groups at the 3-month follow-up.Bold values indicate P < 0.05.

### 3.5 Adverse event outcomes

The overall incidence of adverse events was comparable between the groups, with the exception of gastrointestinal reactions (**Table 5**). The intervention group experienced a significantly lower rate of clinically significant (Grade ≥2) gastrointestinal reactions compared to the control group (4/75 [5.33%] vs. 14/75 [18.67%]; χ² = 6.313, P = 0.012). There were no statistically significant differences in the rates of hepatic dysfunction, myelosuppression, neurotoxicity, or alopecia (all P > 0.05).

**Table 5:** Comparison of adverse event incidence between the two groups (n, %).

Adverse event (Grade ≥2)	Intervention group (n=75)	Control group (n=75)	χ²	P
Gastrointestinal reaction	4 (5.33)	14 (18.67)	6.313	0.012
Hepatic dysfunction	5 (6.67)	7 (9.33)	0.421	0.516
Myelosuppression	4 (5.33)	7 (9.33)	0.883	0.347
Neurotoxicity	4 (5.33)	6 (8.00)	0.429	0.512
Alopecia	8 (10.67)	7 (9.33)	0.074	0.786

## 4 Discussion

This study demonstrated that an evidence-based nursing intervention guided by a quantitative assessment strategy (EB-NQAS) significantly reduced cancer-related fatigue, improved self-management ability and quality of life, and decreased the incidence of gastrointestinal adverse events in lung cancer patients undergoing chemotherapy, when compared to routine nursing care. These findings underscore the value of a structured, proactive, and data-driven approach to supportive oncology nursing.

Chemotherapy is pivotal for eliminating micrometastases and improving prognosis in lung cancer, yet its long-term use and associated adverse effects (e.g., fatigue, nausea) often exacerbate psychological distress (e.g., anxiety, depression), potentially disrupting treatment adherence and reducing QoL (22). Traditional nursing, often lacking systematic assessment and tailored interventions, may be insufficient to fully address these complex needs (23). Our findings align with prior studies showing that evidence-based nursing (EBN) can mitigate chemotherapy-related adverse effects and psychological symptoms by integrating scientific evidence (24). A key component of our intervention, the Edmonton Symptom Assessment Scale (ESAS), has been recognized for its role in quantifying symptoms to guide personalized care and dynamic monitoring (13). This study extends these observations by demonstrating that the systematic combination of ESAS with EBN (i.e., EB-NQAS) not only optimizes symptom management but also strengthens nurse-patient communication and collaboration.

Regarding CRF, our results showed significant reductions in all fatigue domains post-intervention. These findings corroborate prior research showing that structured nursing interventions reduced CRF and improved QoL in advanced lung cancer patients (25). Similarly, studies emphasizing comfort care and structured exercise during chemotherapy have linked symptom-specific interventions to lower CRF levels (26). The EB-NQAS likely achieved this by using ESAS to quantify fatigue severity and then implementing tailored, evidence-based interventions, such as personalized energy conservation plans and goal-oriented light physical activity, thereby empowering patients to manage their fatigue proactively.

For self-management ability, the EB-NQAS group showed marked improvements across all domains. This aligns with prior research indicating that EBN can enhance patients’ capacity to manage symptoms, treatment, and psychosocial challenges (27, 28). This was likely because the EB-NQAS protocol actively engaged patients in their care by educating them on symptom self-monitoring, collaborative goal-setting, and specific self-care techniques, as detailed in our intervention. This structured approach empowered patients to take an active role in their care, thereby improving their confidence and self-management skills.

In terms of QoL, the EB-NQAS group exhibited significantly higher functional scores, consistent with studies showing that comprehensive nursing interventions that alleviate treatment-related symptoms also improve functional status (29, 30). The multifaceted approach of EB-NQAS—combining symptom-specific care (e.g., proactive antiemetic advice, graded pain management) with psychological support and self-management training—likely reduced both physical discomfort and emotional distress, which collectively enhanced overall QoL.

The efficacy of EB-NQAS can be attributed to its integration of two key components: quantitative symptom assessment (ESAS) and evidence-based care planning. ESAS provided real-time, objective data on symptom severity, enabling nurses to move beyond reactive care and instead dynamically adjust interventions. Meanwhile, grounding these interventions in evidence-based protocols ensured that care was standardized, effective, and aligned with best practices. This synergy not only improved symptom control but also likely fostered patient confidence and participation in their care, further enhancing outcomes.

This study’s innovation lies in its holistic, data-driven approach to nursing care. Unlike traditional nursing, which often relies on subjective assessment and reactive management, EB-NQAS uses a validated tool to systematically and proactively identify and address patient-reported symptoms, ensuring that interventions are both timely and personalized. The integration of multi-disciplinary evidence into a clear, actionable nursing protocol sets a precedent for collaborative supportive care models in oncology.

## 5 Limitations

Several limitations should be acknowledged. First, the single-center design may limit the generalizability of our findings. Healthcare practices, patient demographics, and resource availability can vary significantly across different hospitals and regions, potentially affecting the implementation and effectiveness of the EB-NQAS protocol. Second, our study focused exclusively on patients receiving chemotherapy-only regimens. With the increasing use of chemo-immunotherapy combinations as a standard of care worldwide, the applicability of our findings to patients receiving these modern treatments may be limited, and this warrants investigation in future studies. Third, baseline symptom burden was not formally assessed with the ESAS in the control group, which could have provided a more robust comparison of the two arms’ initial symptom profiles, although the groups were well-matched on key demographic and clinical characteristics. Fourth, the intervention is relatively resource-intensive, requiring a dedicated, trained nursing team and regular multidisciplinary meetings. This may pose a barrier to implementation in settings with limited staffing or financial resources. Fifth, due to the nature of the behavioral intervention, neither participants nor care-providing nurses could be blinded to group allocation, which introduces a high risk of performance bias. To mitigate this, outcome assessors were blinded to group assignments, and we used validated, patient-reported outcome measures to standardize data collection. However, participant-reported outcomes could still be influenced by their awareness of being in the intervention group (Hawthorne effect). Sixth, while ESAS is validated, its subjective nature may introduce measurement bias. Finally, the 3-month follow-up period may not be sufficient to assess the long-term durability of the intervention’s effects.

## 6 Future directions

Despite these limitations, our findings support EB-NQAS as a promising intervention for improving outcomes in lung cancer patients undergoing chemotherapy. Future research should prioritize multi-center trials to confirm our results in more diverse populations and healthcare settings. Importantly, the EB-NQAS framework should be adapted and tested in patient populations receiving other systemic therapies, such as chemo-immunotherapy or targeted therapy, to evaluate its effectiveness across different treatment contexts. The integration of a multidisciplinary team, including dietitians, physiotherapists, and psychologists, within the EB-NQAS model could further enhance its holistic impact by providing more specialized and comprehensive care. Extending the follow-up period to 6 or 12 months would also be crucial to assess the long-term sustainability of the observed benefits. Finally, integrating objective biomarkers of fatigue and stress (e.g., inflammatory cytokines) could help elucidate the biological mechanisms underlying the effects of the intervention.

## 7 Conclusion

In conclusion, this randomized controlled trial provides strong evidence that an evidence-based nursing strategy guided by quantitative symptom assessment effectively reduces cancer-related fatigue, enhances self-management ability, and improves quality of life in lung cancer patients undergoing chemotherapy. The EB-NQAS protocol offers a feasible and effective model for delivering structured, proactive, and patient-centered supportive care in the oncology setting.

## Data Availability

The original contributions presented in the study are included in the article/supplementary material. Further inquiries can be directed to the corresponding authors.
